# Molecular characterization of the FCoV-like canine coronavirus HLJ-071 in China

**DOI:** 10.1186/s12917-021-03073-8

**Published:** 2021-11-27

**Authors:** Zhige Tian, Qing Pan, Miaomiao Zheng, Ying Deng, Peng Guo, Feng Cong, Xiaoliang Hu

**Affiliations:** 1 Faculty of Agriculture, Forestry and Food Engineering, Yibin University, Yibin, China; 2 Yibin Key Laboratory of Zoological Diversity and Ecological Conservation, Yibin, 644000 China; 3 State Key Laboratory of Veterinary Biotechnology, Harbin Veterinary Research Institute, Chinese Academy of Agricultural Sciences, Harbin, 150001 People’s Republic of China; 4 Guangdong Laboratory Animals Monitoring Institute and Guangdong Provincial Key Laboratory of Laboratory Animals, Guangzhou, 510633 China

**Keywords:** Canine coronavirus, Transcription-regulating sequence, Recombination, Cell tropism

## Abstract

**Background:**

According to the differences of antigen and genetic composition, canine coronavirus (CCoV) consists of two genotypes, CCoV-I and CCoV-II. Since 2004, CCoVs with point mutations or deletions of NSPs are contributing to the changes in tropism and virulence in dogs.

**Results:**

In this study, we isolated a CCoV, designated HLJ-071, from a dead 5-week-old female Welsh Corgi with severe diarrhea and vomit. Sequence analysis suggested that HLJ-071 bearing a complete ORF3abc compared with classic CCoV isolates (1-71, K378 and S378). In addition, a variable region was located between S gene and ORF 3a gene, in which a deletion with 104 nts for HLJ-071 when compared with classic CCoV strains 1-71, S378 and K378. Phylogenetic analysis based on the S gene and complete sequences showed that HLJ-071 was closely related to FCoV II. Recombination analysis suggested that HLJ-071 originated from the recombination of FCoV 79-1683, FCoV DF2 and CCoV A76. Finally, according to cell tropism experiments, it suggested that HLJ-071 could replicate in canine macrophages/monocytes cells.

**Conclusion:**

The present study involved the isolation and genetic characterization of a variant CCoV strain and spike protein and ORF3abc of CCoV might play a key role in viral tropism, which could affect the replication in monocyte/macrophage cells. It will provide essential information for further understanding the evolution in China.

**Supplementary Information:**

The online version contains supplementary material available at 10.1186/s12917-021-03073-8.

## Background

Coronavirus, belong to family *Coronaviridae*, order *Nidovirales*, is single-stranded positive-sense RNA viruses, which have been widely detected in wild animals [1], domesticated animals [2, 3], humans [4] and pets [5].

Currently, coronaviruses can be divided into 4 subfamily, named Alpha, Beta, Gamma and Delta [6]. Canine coronavirus is a member of alpha subfamily of coronavirus, which based on the spike protein gene was divided into two distinct genotypes, CCoV I and CCoV II [7, 8], both of which were distributed widely [9–14]. There were two different subtypes, CCoV IIa and CCoV IIb, have been found in dogs. CCoV IIa was served as the classic CCoV strains, which caused mild enteritis in young dogs [15]. CCoV IIb was emerged because of the homologous recombination between the transmissible gastroenteritis virus of swine (TGEV) and CCoV IIa strains [16], which caused the acute gastroenteritis and the virus was detected in the guts and internal organs [16]. Intermediate viruses, CCoV-A76 possessing a distinct spike with pathogenicity, which is the result of a recombination between CCoV I and CCoV II, had been detected [17].

Since 2004, more virulent CCoV strains with systemic disease have been reported without obvious coinfections [18–21]. Among of these infections, CB/05 was identified as a pantropic, highly pathogenic variant of CCoV type II which could be detected in the internal organs and caused both enteric and systemic signs [21, 22]. In addition, HLJ-073 causing gross multiple organ lesions and diarrhea had been isolated and identified as a pantropic strain in our laboratory. Sequence analysis suggested that the strain bearing a 350-nt deletion of ORF3abc and originated from the recombination of FCoV 79-1683 and CCoV A76. Cell tropism experiments suggested that HLJ-073 could effectively replicate in canine macrophages/monocytes and human THP-1 cells [21]. Therefore, it needs to concern the switch mechanism of cell tropism for CCoV.

In this study, the isolation of a novel pantropic CCoV strain, HLJ-071, is reported in China, which genetically related to the prototype HLJ-073. To better understand the genetic characterization of a variant CCoV strain, the analysis of complete genome sequences, phylogenetic tree and cell tropism was obtained. It will provide essential information for further understanding the evolution in China.

## Methods

### Clinical case

During the summer of 2015, a dead 5-week-old female Welsh Corgi with severe diarrhea and vomit was submitted for laboratory investigation. Necropsy of the dog showed hemorrhagic enteritis and lung on their surfaces. At post-mortem examination, samples were from intestine, brain, lungs, spleen, liver, kidneys, heart and mesenteric lymph nodes for real-time RT-PCR investigation of CCoV RNA [23].

### Isolation and purification

Samples of intestine, brain, lungs, spleen, liver, kidneys, heart and mesenteric lymph nodes were examined for the major viral pathogens and bacterial. Rapid diagnosis kits were employed to identify general canine viral pathogens, including canine distemper virus (CDV), canine parvovirus (CPV), canine adenovirus-1 (CAdV-1), CAdV-2 and CCoV (Bionote, Hwaseong-si, Gyeonggi-do, South Korea). Major canine bacterial, *Bordetella bronchiseptica*, *Pasteurella multocida*, *Leptospira interrogans* were identified by PCR assay [24]. The primers P-F and P-R for CCoV were employed as described [21] (Table S1).

Crandell feline kidney (CrFK) cells were grown in D-MEM supplemented with 10% foetal calf serum (FCS). The fecal sample was homogenized in phosphate-buffered saline (PBS) and centrifuged at 3,000 g for 15 min. Then the supernatant was filtered through a 0.22-μm-pore-size filter and inoculated into CrFK cells, which were confluent into the monolayers. When the sample was passaged three times, cytopathic effects (CPE) was observed. Then, after three rounds of purification by plaque assay [25], the purified virus was titrated and harvested by one cycle of freezing and thawing, and aliquots were stored at -80°C.

### Electron microscopy

The electron microscopy protocol for negative-stain and thin-section examination was described previously [26, 27].

### Isolation and culture of the canine blood monocytes

Canine blood monocytes were isolated following the previous described [28]. Briefly, canine blood monocytes were isolated from 5 five specific-pathogen-free (SPF) dogs. The blood mononuclear cells were purified on Histopaque-1077 (Sigma-Aldrich), and then seeded in a 24-well dish and cultured at 37 °C with 5% CO_2_. After 24 h, nonadherent cells were removed and washed twice with PBS buffer.

### Growth curve and titrations of HLJ-071

The CrFK cells were infected at multiplicity of infection (MOI) of 0.1. After 1 h of adsorption at 37 °C, cells were washed twice with the PBS buffer and incubated at 37 °C with 5% CO_2_. To determine the growth kinetics of the virus in canine blood monocytes, cells were infected at an MOI of 1, and after 1 h of adsorption at 37 °C, monocytes were gently washed three times with PBS medium to remove residual virus and incubated at 37 °C with 5% CO_2_ [28]. The titers are given as the means from triplicate experiments (log 10 TCID 50 /ml); error bars represent standard deviations.

### Indirect immunofluorescence assay (IFA)

The IFA was conductive by a standard procedure. Briefly, mononuclear cells were inoculated with CCoV HLJ-071 isolates at a MOI of 1 for 48 h and removed the supernatant medium, after washing with PBS, the infected cells were fixed with paraformaldehyde (4%) for 30 min. After blocking with 2% BSA for 2 h at 37 °C, the cells were incubated with N protein polyclonal antibodies (1:400) for 1 h at 37 °C, then the fluorescein isothiocyanate-conjugated goat anti-mouse antibody against immunoglobulin G (1:1000; Abcam, British). The CCoV-N polyclonal antibody was prepared in our laboratory [21].

### Genome sequencing and phylogeny analysis

Fourteen pairs of primers were designed based on the conserved regions of CCoV strain HLJ-073 [21]. The RNA extraction and cDNA synthesis were performed as previously [26].

Sequence data were assembled and analyzed using Clustal X software (1.83), Vector 10 and DNASTAR. Phylogenetic trees based on the complete sequences and the spike proteins were performed using Neighbor-joining (NJ) method with Kimura 2-parameter model in molecular evolutionary genetics analysis software (version 4.0). The support for the tree nodes was calculated with 1,000 replicates. Simplot 3.5.1 was conductive for evaluating the recombination events between the reference CCoV and FCoV strains. The HLJ-071 sequence obtained in this study was assembled and submitted to the GenBank database under accession number KY063616.

## Recults

### Viral isolation and identification

**Fig. 1:**
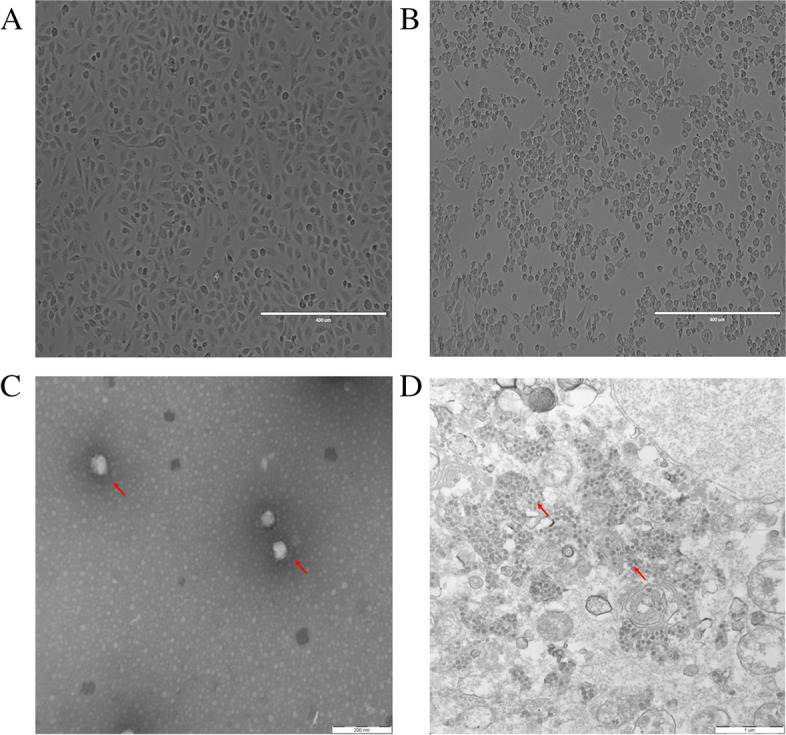
**A** control (uninfected) CrFK cells. **B** Cytopathic effect (CPE) induced by HLJ-071 in the CrFK cells. **C** Electron micrograph of HLJ-071 negatively stained with 2% phosphotungstic acid. The scale bar represents 200 nm. **D** ultra-thin sections of infected CrFK cells with HLJ-071 displayed the typical particles in the cytosol

The results of Colloidal gold diagnostic reagent and PCR confirmed that the fecal sample was CCoV-positive; the sample was negtive for CPV, CAV, CDV and major canine bacterials (data not shown). All of the organs except the heart were found to be positive for CCoV (Table S2), indicating that this strain is a pantropic CCoV strain. After inoculation of CrFK cells with samples and three serial passages, one CCoV isolate designated that HLJ-071 was successfully obtained from the fecal samples and CPE were found in the CrFK cells at 3-5 days post-inoculation with rounding and the detachment of the cells into the medium (Fig. 1B). The titre of HLJ-071 was 10^7.5^ TCID_50_/mL in CrFK cells. Electron microscopy observed that the virus displayed a circular shape with petal-shaped, which had diameters of about 150 nm. Ultra-thin sections of infected CrFK cells displayed typical virus particles in the cytoplasm (Fig. 1D).

### Full-length nucleotide sequence and phylogenetic analysis

The complete genome sequence of HLJ-071 was assembled and comparative analysis with other canine coronavirus was performed. The full genome of HLJ-071 was amplified using the 14 pairs of primers referenced to HLJ-073. The complete sequence of HLJ-071 was 29,319 nucleotides (nts) in length, including 5’non-translated region (NTR)-ORF1-S-ORF3abc-E-M-N-ORF7ab-3’poly A tail. The 5’portion NTR of the genome contained a 230-nt NTR, ORF1a (231-12,287) and ORF1ab (231-20,057). Four structural proteins S, E, M and N were found to be encoded by ORF S (20,284-24,648 nt), ORF E (25,826-26,074 nt), ORF M (26,055-26,876 nt) and ORF N (26,889-28,037 nt). Five non-structural protein-coding genes were ORF3a (24,712-24,948 nt), ORF3b (24,893-25,108 nt), ORF3c (25,105-25,860 nt), ORF7a (28,042-28,347 nt) and ORF7b (28,352-28,993 nt).

**Fig. 2:**
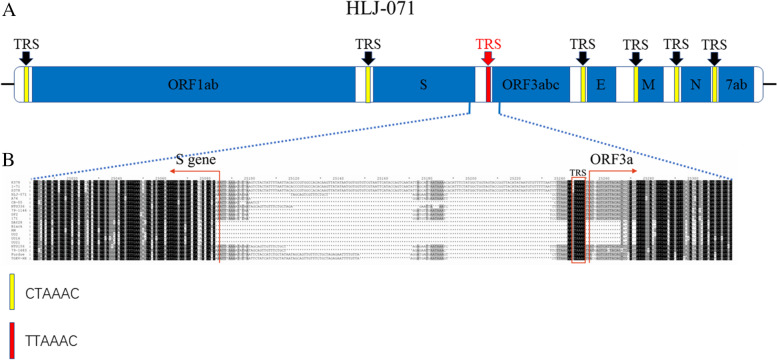
Schematic representation of genome and TRS of the HLJ-071 (**A**) and Multiple sequence alignment of the partial S and ORF3a of CCoV strains (**B**)

Sequence analysis suggested that there was an entire ORF3abc with 1,149 nts when compared to classic CCoV strains 1-71 and K378. Interestingly, comparing with classic CCoV strains 1-71, S378 and K378, there is a variable region was located between S gene and ORF 3a gene in varient CCoV strains (HLJ-071, A76 and CB/05). For HLJ-071, there is a 104-nt deletion in that region (Fig. 2). A highly conserved core sequence transcription regulatory sequences (TRS) is 5’-CTAAAC-3’ in CCoV. However, a unique mutant TRS, 5’-UUAAAC-3’, was presents in classic 1-71, K378, S378 and located between gene of S and ORF3a, comparing with HLJ-071, varients CCoV, FECV, FIPV and TGEV strains (Fig. 2), which may influence the stability of RNA transcription and expression of subgenome.

**Fig. 3:**
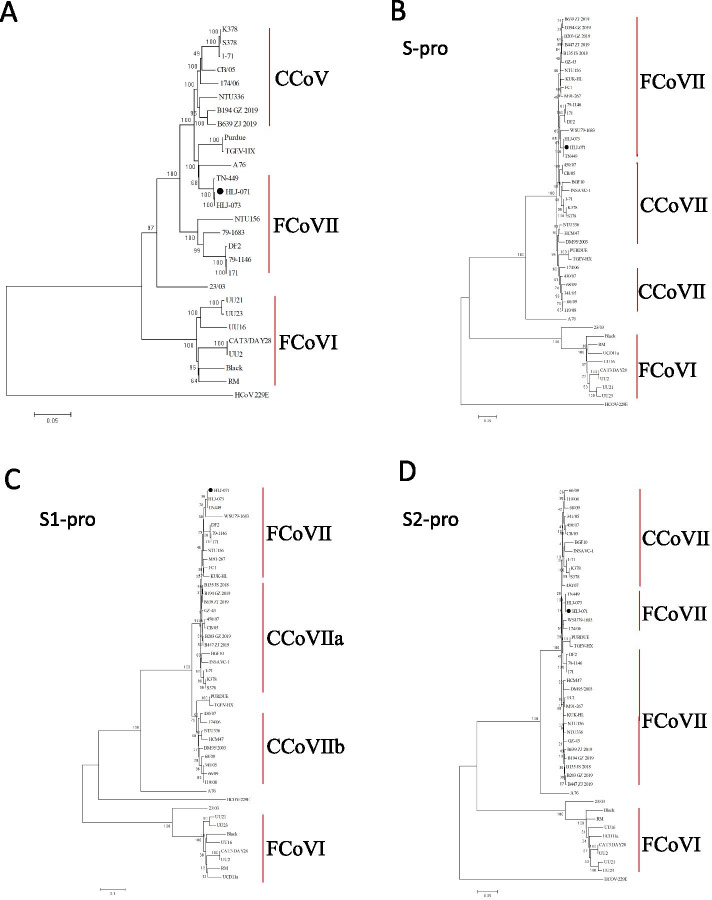
Phylogenetic analysis of the complete sequences, spike protein (S), S1, S2, genome regions of HLJ-071. Neighbor-joining was used for the construction of the phylogenetic tree with bootstrap values of 1000 replicates shown at the branches. The scale bar represents the p-distance

**Fig. 4:**
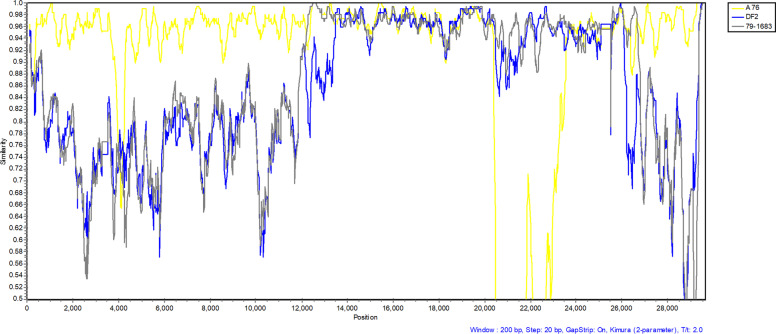
Similarity plot of the compelte genome nucleotide sequence of CCoV HLJ-071 and three reference CCoV strains, FCoV DF2, FCoV WSU79-1683 and CCoV A76. The other parameters used included the Kimura (2-parameter) distance model, 2.0 Ts/Tv ratio, neighbor-joining tree model, and 1000 bootstrap replicates

By phylogenetic analysis, HLJ-071 based on complete sequences show that divided into FCoVs cluster, closed with TN-449, HLJ-073 and A76. The complete spike protein did not cluster with either type I or type II CCoVs, and related to FCoV WSU 79-1683. In addition, analysis of S1 (receptor-binding) domian showed that it clustered closely with FCoV 79-1683 and HLJ-073, while S2 (fusion) domain clustered with CCoV IIb 174/06 and WSU79-1683 (Fig. 3). The occurrence of recombination of HLJ-071 between CCoV A76 and FCoV 79-1683 and FCoV DF2 was detected, which had led to a new genetype emergence of the FCoV-like CCoVs (Fig. 4).

### Cell tropism of HLJ-071

**Fig. 5:**
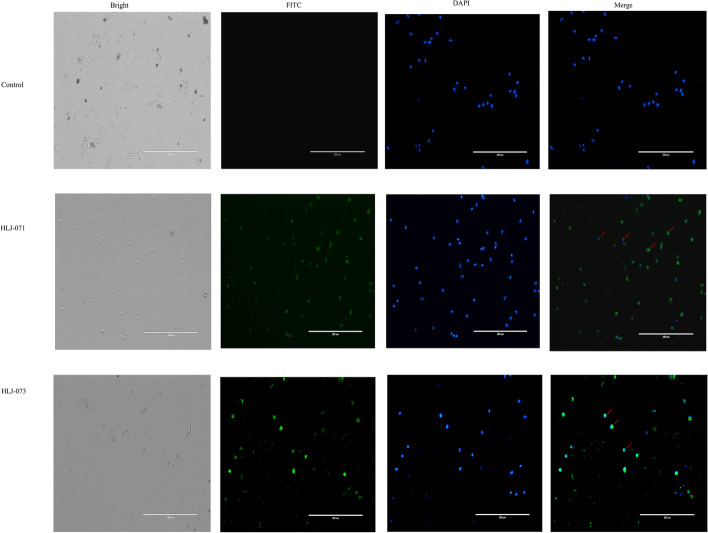
Indirect immunofluorescence detection of CCoVs in canine macrophage/monocyte. The cells were infected with HLJ-071 or HLJ-073 (MOI = 1) and detected using CCoV N protein-positive serum

**Fig. 6:**
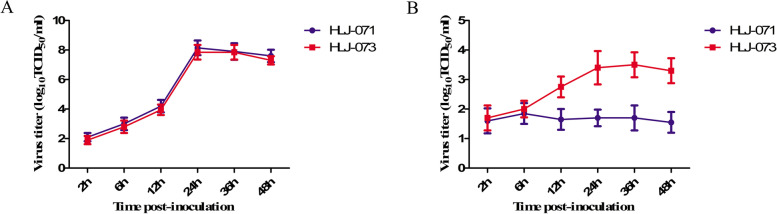
Replication dynamics of the HLJ-071 and HLJ-073. Growth kinetics of the HLJ-071 and HLJ-073 after infection of CrFK (**A**) (MOI of 0.1) and canine macrophage/monocyte cells (**B**) (MOI of 1). The titers are given as the means from triplicate experiments (log 10 TCID 50 /ml); error bars represent standard deviations

Previous studies showed that the ORF3abc deletion of canine and feline coronavirus alter the cell tropism [21, 28]. To further investigate the *in vitro* growth characteristics of HLJ-071, canine monocytes cells were inoculated with HLJ-071 and HLJ-073 at an MOI of 1. The results showed that HLJ-073 could efficiently replicate in canine monocytes, however, HLJ-071 could poorly replicate in that cells (Fig. 5). Furthmore, the titers of HLJ-071 and HLJ-073 were determined at 24 h p. i. in CrFK cell lines and canine monocytes cells. The results showed that growth characteristics of HLJ-071 was similar to HLJ-073 and reached the 10^7.5^ TCID_50_/mL at 24 h p.i. in CrFK cell lines (Fig. 6A). However, the titers of HLJ-071 peaked at 2×10^1.6^ TCID_50_/mL at 6 h p.i. significantly slower growth characteristics compared to titers of HLJ-073 reached at 10^3.5^ TCID_50_/mL at 36 h p.i. (Fig. 6).

## Discussion

Generally, CCoV mainly causes intestinal infections, resulting in the viral enteritis and diarrhea in dog polulation. In the last decades, an increasing number of pantropic strains had been reported with system infections, causing multiple organ damage [29]. However, the reason why the tissue tropism of CCoVs changed from enteropathogenic to system infection is unknown.

In this study, we isolated a CCoV, HLJ-071, from a dead 5-week-old female Welsh Corgi without apparent coinfections. Phylogenetic analysis suggested that HLJ-071 based on the complete sequence was closed to FCoV II and distincted with other CCoV I and II strains. In terms of the major structural protein spike protein, HLJ-071 was closely related to FCoV WSU79-1683 and domestic strain HLJ-073, different from other Chinese strains B135/JS/2018, B194/GZ/2019, B639/ZJ/2019, B203/GZ/2019 and B447/ZJ/2019. HLJ-071 with HLJ-073 and TN-449 was formed an unique cluster between the classical CCoVs and FCoVs. All of these suggested that domestic strains were derived from the different ancestor and co-circulated in China and underwent evolution.

The cell tropism of FCoVs, so far, has been well investigated. Previous studies indicated that the spike protein and ORF3abc played a crucial part in macrophae infection in FCoVs [28, 30]. For feline infectious peritonitis viruses (FIPV), the virus could replicate in the monocyte/macrophage lineage cells and then disseminate that to the organs causing system infections [31]. On the country, feline enteric coronavirus (FECV) was primary replicated in enterocytes and could not replicate in the monocyte/macrophage lineage cells. For CCoV, a system infection with CCoV CB/05 with a partial detetion of ORF3b and a FCoV-like spike protein was found in 2005 [32]. We speculated that stain could replicate in monocyte/macrophage cells, although no available data in viral tropism experiment. Furthmore, we have isolated and reported a CCoV strain, HLJ-073 bearing a 350-nt deletion in ORF3abc and a FCoV-like spike protein, could efficient replicate in canine monocyte/macrophage cells and human THP-1 cells [21]. In this study, HLJ-071 bearing entire ORF3abc and FCoV spike protein could weakly replicate in canine monocyte/macrophage cells comparing with HLJ-073, which was distinct with the cell tropism of FECV. These suggested that 1) both of HLJ-071 and HLJ-073 had a common feature, that were the acquissiton of macrophage tropism; 2) ORF3abc could enhance the replication in monocyte/macrophage cells, when compared with HLJ-071 and HLJ-073.

Previous studies indicated that 5’ and 3’ flanks of the TRSs of TGEV and MHV influenced transcription levels (accumulation) and protein expression [33, 34]. Besides, the regions flanking the TRS also have a profound impact on the production of subgenomic RNA [35, 36]. In this study, a mutant TRS was found in classic CCoVs 1-71, S378, K378 and a variable region was discoved in varient CCoVs HLJ-071. Both of these suggested that 1) the transcription levels and protein expression of ORF3abc were affected, and 2) ORF3abc might be associated with an alternation in virus tropism. Further research was needed to investigate the relationship between transcription of ORF3abc or the pathogenicity of CCoV with these variable regions and mutant TRS.

## Conclusion

In this present, we indicated that 1) CCoV HLJ-071 was closely related to FCoVs and recombinated with CCoVs and FCoVs, indicated that CCoVs underwent a rapid evolution in China. 2) The cell tropism of CCoVs may be correlated with the function ORF3abc and transcription of subgenome.

## Supplementary Information

**Additional file 1: Table S1.** Primers used for identifying and completely sequencing the strains

**Additional file 2: Table S2.** RNA copies (log_10_(CoV genome copies per 10^3^ GAPDH copies)) of template in the samples of dead puppy, tested by specific real time RT-PCR

## Data Availability

The complete sequences obtained in this study have been submitted to the GenBank database (accession number: KY063616).

## Abbreviations

CAdV-1: Canine adenovirus-1
CDV: Canine distemper virus
CPV: Canine parvovirus
CrFK: Crandell feline kidney
CPE: Cytopathic effects
FECV: Feline enteric coronavirus
FIPV: Feline infectious peritonitis viruses
FCS: Foetal calf serum
IFA: Indirect immunofluorescence assay
MOI: Multiplicity of infection
NJ: Neighbor-joining
NTR: Non-translated region
SPF: Specific-pathogen-free
TRS: Transcription regulatory sequences
TGEV: Transmissible gastroenteritis virus of swine

## References

[1] Yang XL, Hu B, Wang B, Wang MN, Zhang Q, Zhang W, Wu LJ, Ge XY, Zhang YZ, Daszak P (2015). Isolation and characterization of a novel bat coronavirus closely related to the direct progenitor of severe acute respiratory syndrome coronavirus. J Virol.

[2] Jung K, Saif LJ, Wang Q (2020). Porcine epidemic diarrhea virus (PEDV): An update on etiology, transmission, pathogenesis, and prevention and control. Virus Res.

[3] McCluskey BJ, Haley C, Rovira A, Main R, Zhang Y, Barder S (2016). Retrospective testing and case series study of porcine delta coronavirus in U.S. swine herds. Prev Vet Med.

[4] Morfopoulou S, Brown JR, Davies EG, Anderson G, Virasami A, Qasim W, Chong WK, Hubank M, Plagnol V, Desforges M (2016). Human Coronavirus OC43 Associated with Fatal Encephalitis. N Engl J Med.

[5] Tekes G, Thiel HJ (2016). Feline coronaviruses: pathogenesis of feline infectious peritonitis. Adv Virus Res.

[6] Carstens EB (2010). Ratification vote on taxonomic proposals to the International Committee on Taxonomy of Viruses (2009). Arch Virol.

[7] Decaro N, Buonavoglia C (2008). An update on canine coronaviruses: viral evolution and pathobiology. Vet Microbiol.

[8] Le Poder S (2011). Feline and canine coronaviruses: common genetic and pathobiological features. Adv Virol.

[9] Costa EM, de Castro TX, Bottino Fde O, Garcia Rde C (2014). Molecular characterization of canine coronavirus strains circulating in Brazil. Vet Microbiol.

[10] Cavalli A, Desario C, Kusi I, Mari V, Lorusso E, Cirone F, Kumbe I, Colaianni ML, Buonavoglia D, Decaro N (2014). Detection and genetic characterization of Canine parvovirus and Canine coronavirus strains circulating in district of Tirana in Albania. J Vet Diagn Investig.

[11] Licitra BN, Whittaker GR, Dubovi EJ, Duhamel GE (2014). Genotypic characterization of canine coronaviruses associated with fatal canine neonatal enteritis in the United States. J Clin Microbiol.

[12] Pratelli A, Decaro N, Tinelli A, Martella V, Elia G, Tempesta M, Cirone F, Buonavoglia C (2004). Two genotypes of canine coronavirus simultaneously detected in the fecal samples of dogs with diarrhea. J Clin Microbiol.

[13] Decaro N, Mari V, Elia G, Lanave G, Dowgier G, Colaianni ML, Martella V, Buonavoglia C (2015). Full-length genome analysis of canine coronavirus type I. Virus Res.

[14] Wang X, Li C, Guo D, Wang X, Wei S, Geng Y, Wang E, Wang Z, Zhao X, Su M (2016). Co-circulation of canine coronavirus I and IIa/b with high prevalence and genetic diversity in Heilongjiang Province, Northeast China. PLoS One.

[15] Decaro N, Buonavoglia C (2011). Canine coronavirus: not only an enteric pathogen. Vet Clin North Am Small Anim Pract.

[16] Decaro N, Mari V, Campolo M, Lorusso A, Camero M, Elia G, Martella V, Cordioli P, Enjuanes L, Buonavoglia C (2009). Recombinant canine coronaviruses related to transmissible gastroenteritis virus of Swine are circulating in dogs. J Virol.

[17] Regan AD, Millet JK, Tse LP, Chillag Z, Rinaldi VD, Licitra BN, Dubovi EJ, Town CD, Whittaker GR (2012). Characterization of a recombinant canine coronavirus with a distinct receptor-binding (S1) domain. Virology.

[18] Decaro N, Martella V, Elia G, Campolo M, Desario C, Cirone F, Tempesta M, Buonavoglia C (2007). Molecular characterisation of the virulent canine coronavirus CB/05 strain. Virus Res.

[19] Zappulli V, Caliari D, Cavicchioli L, Tinelli A, Castagnaro M (2008). Systemic fatal type II coronavirus infection in a dog: pathological findings and immunohistochemistry. Res Vet Sci.

[20] Escutenaire S, Isaksson M, Renström LH, Klingeborn B, Buonavoglia C, Berg M, Belák S, Thorén P (2007). Characterization of divergent and atypical canine coronaviruses from Sweden. Arch Virol.

[21] Chen S, Liu D, Tian J, Kang H, Guo D, Jiang Q, Liu J, Li Z, Hu X (2019). Molecular characterization of HLJ-073, a recombinant canine coronavirus strain from China with an ORF3abc deletion. Arch Virol.

[22] Ntafis V, Mari V, Decaro N, Papanastassopoulou M, Papaioannou N, Mpatziou R, Buonavoglia C, Xylouri E (2011). Isolation, tissue distribution and molecular characterization of two recombinant canine coronavirus strains. Vet Microbiol.

[23] Decaro N, Pratelli A, Campolo M, Elia G, Martella V, Tempesta M, Buonavoglia C (2004). Quantitation of canine coronavirus RNA in the faeces of dogs by TaqMan RT-PCR. J Virol Methods.

[24] Gravekamp C, Van de Kemp H, Franzen M, Carrington D, Schoone GJ, Van Eys GJ, Everard CO, Hartskeerl RA, Terpstra WJ (1993). Detection of seven species of pathogenic leptospires by PCR using two sets of primers. J Gen Microbiol.

[25] Tuchiya K, Kasaoka T, Azetaka M, Takahashi E, Konishi S (1987). Plaque assay for canine coronavirus in CRFK cells. Nihon Juigaku Zasshi Japan J Vet Sci.

[26] Hu X, Li N, Tian Z, Yin X, Qu L, Qu J (2015). Molecular characterization and phylogenetic analysis of transmissible gastroenteritis virus HX strain isolated from China. BMC Vet Res.

[27] Li Z, Shao Y, Liu C, Liu D, Guo D, Qiu Z, Tian J, Zhang X, Liu S, Qu L (2015). Isolation and pathogenicity of the mammalian orthoreovirus MPC/04 from masked civet cats. Infect Genet Evol.

[28] Bálint Á, Farsang A, Zádori Z, Hornyák Á, Dencso L, Almazán F, Enjuanes L, Belák S (2012). Molecular characterization of feline infectious peritonitis virus strain DF-2 and studies of the role of ORF3abc in viral cell tropism. J Virol.

[29] Buonavoglia C, Decaro N, Martella V, Elia G, Campolo M, Desario C, Castagnaro M, Tempesta M (2006). Canine coronavirus highly pathogenic for dogs. Emerg Infect Dis.

[30] Rottier PJ, Nakamura K, Schellen P, Volders H, Haijema BJ (2005). Acquisition of macrophage tropism during the pathogenesis of feline infectious peritonitis is determined by mutations in the feline coronavirus spike protein. J Virol.

[31] Kipar A, May H, Menger S, Weber M, Leukert W, Reinacher M (2005). Morphologic features and development of granulomatous vasculitis in feline infectious peritonitis. Vet Pathol.

[32] Decaro N, Mari V, von Reitzenstein M, Lucente MS, Cirone F, Elia G, Martella V, King VL, Di Bello A, Varello K (2012). A pantropic canine coronavirus genetically related to the prototype isolate CB/05. Vet Microbiol.

[33] Alonso S, Izeta A, Sola I, Enjuanes L (2002). Transcription regulatory sequences and mRNA expression levels in the coronavirus transmissible gastroenteritis virus. J Virol.

[34] Zhang X, Liu R (2000). Identification of a noncanonical signal for transcription of a novel subgenomic mRNA of mouse hepatitis virus: implication for the mechanism of coronavirus RNA transcription. Virology.

[35] Sawicki SG, Sawicki DL, Younker D, Meyer Y, Thiel V, Stokes H, Siddell SG (2005). Functional and genetic analysis of coronavirus replicase-transcriptase proteins. PLoS Pathog.

[36] Zúñiga S, Sola I, Alonso S, Enjuanes L (2004). Sequence motifs involved in the regulation of discontinuous coronavirus subgenomic RNA synthesis. J Virol.

