# Supplementary material for: Molecular characterization of the FCoV-like canine coronavirus HLJ-071 in China
Source: BMC Vet Res. 2021 Nov 27;17:364. doi: 10.1186/s12917-021-03073-8 (PMC8626285; doi:10.1186/s12917-021-03073-8)
Supplement: Supplementary file 1 — Additional file 1: Table S1. Primers used for identifying and completely sequencing the strains [file 12917_2021_3073_MOESM1_ESM.docx]

Table S1. Primers used for identifying and completely sequencing the strains

| Names | Sequences | Positions |
| --- | --- | --- |
| 1F | TCCAGATATGTAATGTTCGG | 26498-26517 |
| 1R | TCTGTTGAGTAATCACCAGCT | 26887-26907 |
| 2F | TAGTACAGAAAATTTAGACCC | 23144-23164 |
| 2R | AAGTACAAATGTAATAATTGC | 26534-26554 |
| 3F | GGATGGGCTTACTATGTAAAA | 26861-26881 |
| 3R | ACTAAATCTAGCATTGCCAAA | 29211-29231 |
| 4F | AGCGTGGCTATCTCTCTTCTT | 23-43 |
| 4R | GTTTTGAAAGTAGAAAATGGT | 2641-2661 |
| 5F | AATAAGCTCTGTAATGCTGCT | 2594-2614 |
| 5R | GTCTCTGTAATGCAAGACAAA | 5091-5111 |
| 6F | CTTCATTGTACATAAGCAGAC | 5044-5064 |
| 6R | CATCATAATTTGTTTAAGCTG | 7505-7525 |
| 7F | GATCTTCCTTATGAAAGATTC | 7433-7453 |
| 7R | ATCTAGTAGTTTCTCAACACT | 9719-9739 |
| 8F | CAAAACTAACAGTTTCACAGA | 9649-9669 |
| 8R | GTTTACCAGCTTGCAGACGAA | 11895-11915 |
| 9F | TTAGACGTGGTGCAGTTCTTG | 11856-11876 |
| 9R | GTAACAACAACTTCACGAGCG | 13767-13787 |
| 10F | ATTTGTTTATAAAATAGTTGG | 13710-13730 |
| 10R | TAAGGCACCAGTGTATTATAG | 15882-15902 |
| 11F | TACCATATCTAAGCTCTATCC | 15840-15860 |
| 11R | TCAGAGCAGCTTTCATGACAT | 17834-17854 |
| 12F | TTATTGACAACGAAGAAAAGA | 17783-17803 |
| 12R | GTGTCTAGGACTGAGTTGTGT | 20190-20210 |
| 13F | AAATATAATGCATGCCAATTA | 20133-20153 |
| 13R | ATTGTCCCATGAAGAACTTTT | 22128-22148 |
| 14F | GTTCGTTCTAATCAATTCTCA | 22086-22106 |
| 14R | GGGGTCAAATAGAACTTGCCA | 24164-24184 |
| 3' race | GTTGGTTGTCGAACATCTTTC | 28828-28848 |
| R-F | CCTTAAGAACTAAACTTATGA | 24778-24798 |
| R-R | ATGCCGACACAAGTCTTAAAG | 25400-25420 |
| 5’ race | CGGTAAGAAGAACAGGTTTAA | 445-465 |
