# Supplementary material for: Molecular characterization of the FCoV-like canine coronavirus HLJ-071 in China
Source: BMC Vet Res. 2021 Nov 27;17:364. doi: 10.1186/s12917-021-03073-8 (PMC8626285; doi:10.1186/s12917-021-03073-8)
Supplement: Supplementary file 2 — Additional file 2: Table S2. RNA copies (log10(CoV genome copies per 103 GAPDH copies)) of template in the samples of dead puppy, tested by specific real time RT-PCR [file 12917_2021_3073_MOESM2_ESM.docx]

Table S2. RNA copies (log_10_(CoV genome copies per 10^3^ GAPDH copies)) of template in the samples of dead puppy, tested by specific real time RT-PCR

| Sample | RNA copies |
| --- | --- |
| Faces | 3.95 |
| Lung | 2.57 |
| Intestine | 3.68 |
| Heart | n.d. |
| Liver | 2.04 |
| Spleen | 2.79 |
| Kidneys | 1.78 |
| mesenteric lymph nodes | 2.97 |

n.d., not detected
